# Supplementary material for: Upper extremity kinematics: development of a quantitative measure of impairment severity and dissimilarity after stroke
Source: PeerJ. 2023 Dec 8;11:e16374. doi: 10.7717/peerj.16374 (PMC10712307; doi:10.7717/peerj.16374)
Supplement: Supplemental Information 1 [file peerj-11-16374-s001.pdf]

1. Project Title

Non-invasive brain stimulation to enhance performance and learning of reaching tasks in individuals with stroke induced arm impairment

2. Principal Investigator

Michelle Harris-Love, PT, PhD
Neuroscience Research Center
MedStar National Rehabilitation Hospital
102 Irving Street, N.W.
Washington D. C., 20010
Phone: 202-877-1558
Fax: 202-726-7521
E-mail: Michelle.L.Harris-Love@medstar.net

Additional Personnel Involved in Study

Alexander W. Dromerick, MD
Neuroscience Research Center
MedStar National Rehabilitation Hospital

Evan Chan, MS
Neuroscience Research Center
MedStar National Rehabilitation Hospital

Peter Turkeltaub, MD, PhD
Assistant Professor of Neurology
Department of Neurology
Georgetown University Medical Center

Rachael Harrington, MA, CCC-SLP
Interdisciplinary Program in Neuroscience
Georgetown University

Clinton Wutzke, PhD
VA Research Fellow, Washington, DC

Shashwati Geed, PhD
ARRT Research Fellow
MedStar National Rehabilitation Hospital/Georgetown University

Amanda Rounds, PhD Student
George Mason University

Seth Kiser, PhD
VA Research Fellow, Washington, DC

Pallavi Samudrala
Nova Southeastern University

Sophia Brumme
Catholic University of America

3. Study Location

Study-related activities will take place at MedStar National Rehabilitation Hospital. The expected date for initiation is 2/1/2014. We expect the study to be completed by 01/31/2016.

4. Background

Stroke is a leading cause of serious long-term disability and its prevalence is expected to increase dramatically as the population ages (Lozano et al 2012; Ovbiagele et al 2013). Because arm impairment is a major contributor to stroke-related disability (Broeks et al 1999; Desrosiers et al 2006; Likhi et al 2013), reducing arm impairment could result in enormous benefits (and cost savings) when multiplied across this large and growing population. The mechanisms of arm recovery in mildly impaired patients, who retain partial hand function, have been studied and treatments developed for this group have shown efficacy (Wolf et al 2008). In contrast, little is known about recovery mechanisms in more severely impaired patients (who retain partial arm function but do not have voluntary hand function), and no effective treatments exist. The contribution of the proposed research is expected to be the identification of the role of the intact hemisphere in arm recovery of patients with severe arm impairment. This contribution will be significant because it will open the door to enhanced understanding of recovery mechanisms in stroke patients with severe arm impairment, who make up a large portion of the stroke population and for whom there are no effective treatments. More broadly, it will establish that patterns of neural reorganization after stroke can differ based on individual patient characteristics, and patterns that are beneficial in one sub-group may be deleterious in another. Ultimately, it will provide the foundational work needed to make informed choices of therapeutic targets for interventions, such as non-invasive brain stimulation, that will be based on individual patient profiles and could lead to far greater recovery levels than currently possible.

Non-invasive brain stimulation to enhance arm recovery in stroke patients has had mixed success and has been applied primarily in mildly impaired patients. Many of these approaches have been based on previous studies showing that intact hemisphere activity during affected hand movement is associated with poorer recovery (Ward 2011; Ward & Cohen 2004). Further, the "interhemispheric competition" conceptual model of recovery (Nowak et al 2009; Ward & Cohen 2004) supports the notion that intact hemisphere activation can produce excessive transcallosal inhibition of the stroke-affected hemisphere. Hence, intact hemisphere activation is often considered detrimental to recovery and brain stimulation approaches have largely focused on suppressing it.

However, information gained about recovery mechanisms in mildly impaired patients may not directly translate to those with more severe impairments. The networks and processes that contribute to recovery in patients with significant affected-hemisphere damage are likely to differ from those in patients with more sparing of affected-hemisphere structure and function. The observed correlation between intact hemisphere activation and poorer motor recovery does not imply that intact hemisphere activity causes poorer motor recovery. In some cases, it may

support the residual motor function that exists in patients with more severe strokes (e.g. Johansen-Berg et al 2002). In fact, we know that the intact hemisphere could, in theory, substantially support motor function of the affected arm. When a complete hemispherectomy is performed at a young age, individuals often recover almost full function of the arm ipsilateral to the intact hemisphere (Burke et al 2012; Choi et al 2010; Honda et al 2010), providing striking evidence that, at least early in life, a single cortical hemisphere can control both arms.

Patients with severe arm impairment can make small improvements in reaching with practice, but it is not known if these practice-induced improvements could be enhanced by pre-practice modulation of brain excitability. If the brain areas that can contribute to recovery could be "primed" prior to practice, the effects of practice may be enhanced, and patients could potentially reap greater rewards for their efforts.

5. Study Objective and hypothesis

The objective of this study is to determine the extent to which practice-induced improvements in reaching could be enhanced by pre-practice "priming" of intact hemisphere motor areas.

We will achieve this objective by testing the hypothesis that 1) excitatory TMS to intact dorsal premotor cortex (PMd) will enhance practice effects more than excitatory TMS to intact primary motor cortex (M1), 2) patients with the greatest impairment at baseline will make the largest practice-induced improvements with excitatory TMS to PMd prior to practice, and 3) enhanced effects of practice with excitatory TMS to PMd will be linked to an enhanced role of PMd in reaching movements.

6. Protocol Design

a. Procedures

<u>In-person Screening, Informed Consent and Familiarization with Reaching</u>
Prior to the experiment, all volunteers will be informed of the purpose of the experiment, the complete procedures, and any potential risks associated with participation in the study. There will be no time urgency to the consenting process, and volunteers will be invited to consider participation at their leisure and in consultation with their family, friends, and/or healthcare providers. Once the volunteer has decided to participate in the study, he/she will sign the consent form and the project will begin. One copy of the signed consent form will be provided to the participant; the other will be kept confidential in locked file cabinets located in locked offices.

For the purposes of scientific presentations and publications, the participant will have the option to sign a photo/video release form which authorizes the study personnel to use the participant's photographic or video images, either as originally recorded or altered to obscure their identity (based on their preference). Participants will not be required to sign the photo/video release form in order to participate in the study.

For stroke survivors whose medical records are from sources outside the MedStar network, a medical release form will be prepared in case we need to contact the participants' health care providers to obtain further diagnosis-related information and medical records.

Stroke survivors will also be familiarized with the reaching task by practicing the reaching task with his/her affected arm for at least 20 repetitions. Participants who are unable to complete the task will be withdrawn from the protocol (see exclusion criteria) and will not undergo any further testing.

Stroke survivors who remain eligible for the study will then undergo clinical measures, which include: i.) Upper Extremity Fugl-Meyer (UEFM) test of post-stroke motor impairment, ii.) Mini Mental Status Exam (MMSE) cognitive questionnaire, and iii.) Modified Ashworth scale for spasticity.

Neurological Exam and (Optional) Anatomical MRI

For stroke survivors, a brief neurological exam will be conducted by a study physician. The physician will also review the participant's available medical history including radiological report and stroke history to confirm that he/she meets the study inclusion criteria.

For both stroke survivors and healthy volunteers, whenever possible, a brief, high resolution, anatomical, T1-weighted magnetic resonance imaging (MRI) brain scan without contrast, will be obtained at the Washington Hospital Center (WHC) imaging center. A questionnaire verifying that the participant has no contraindications to MRI will be administered before the scan. This scan is collected for use with the Brainsight™ neuro-navigation system (see below). It is ideal to have a scan of the individual's own neuroanatomy, but in the absence of such a scan, it is still possible to use a "dummy" scan to ensure the repeatability of the stimulation location. Therefore, if there are no contraindications to MRI, yet the participant is unwilling to have the scan, they will still be allowed to proceed with the study.

Results from the neurological exam and images from the anatomical MRI may be reused for participants who have previously participated in related studies, provided that these procedures were administered within the preceding two years and that there have been no additional changes in the participant's neurological status.

Reaching Practice and Testing with iTBS

Once the physical exam and the MRI confirm that the subject remains eligible for further testing, the testing will be conducted over the course of approximately 9, but no more than 12, testing sessions (depending on the participant's fatigability, the emergence of technical problems, or other factors). All participants will perform reaching practice and testing with iTBS applied over up to six cortical sites. The basic testing schedule is summarized in the diagram below. For each cortical site, iTBS will be followed by repetitive reaching practice, along with testing of reaching performance with or without TMS-disruption. Each practice session will be separated by at least one week to reduce carry-over effects.

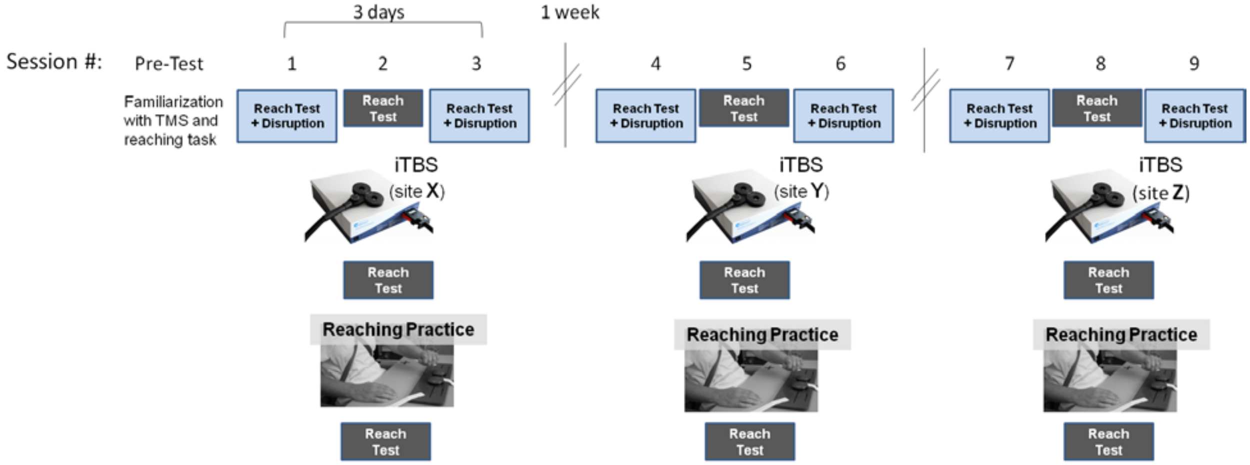

For reaching testing and practice, participants will be asked to respond to a "GO" signal by moving their hand forward to contact a button as quickly as possible. The reaching testing and practice will be organized into short bouts with rest periods between bouts. Small surface electromyography (EMG) electrodes and motion sensors will be placed on the arms during the reaching testing and practice. Reaching response time will be calculated as the time from the "GO" signal to the time the button is pressed. Other measures of reaching performance will include the level of muscle activation (measured via EMG) during reaching and the velocity and smoothness of the reach (measured via the motion sensors).

For iTBS application, a transcranial magnetic stimulator (MagPro X100 with MagOption, MagVenture Inc., Atlanta, GA) will be used to deliver low-intensity stimulation in 20 trains of 10 TMS bursts, delivered at 5 Hz, with each train separated by 8-s intervals, for a total of 200 seconds (3.3 minutes). The stimulation intensity will be below that required to elicit a motor evoked potential in the actively contracting muscle (i.e. below "active motor threshold"). Each TMS burst consists of 3 TMS pulses (delivered at 50 Hz), resulting in 600 TMS pulses given for each cortical site. This is a standard iTBS protocol that has been used in many studies of stroke patients (Ackerley et al 2010; Di Lazzaro et al 2008; Di Lazzaro et al 2010; Hsu et al 2013; Talelli et al 2007; Talelli et al 2012). An iTBS-induced increase in cortical excitability has been reported to last 30-45 minutes after the completion of the stimulation period.

To test for a change in a brain area's contribution to reaching performance when it has been stimulated prior to practice, we will test reaching with TMS-disruption of that brain area before and after the iTBS + reaching practice intervention. To produce a momentary disruption of the firing pattern of the targeted brain area, TMS pulses will be applied to the target brain area at precise time points during the reaction time period (before movement onset). No long-lasting effect is expected with this TMS-disruption paradigm. Trials with TMS pulses will be intermixed with no-TMS trials. To determine whether the targeted area had a functional role in the task at the time the pulses were delivered, we will compare the reaching reaction time in trials with vs. without TMS pulses.

For precise and repeatable positioning of the TMS stimulator, we will use the Brainsight™ neuro-navigation system (Rogue Research Inc., Montreal Quebec, Canada). The participant's anatomical MRI (or a dummy scan if the participant's MRI is unavailable) is loaded into the Brainsight™ software and reflective position markers are placed on the TMS coil and on a padded headband or glasses which the participant wears. Using anatomical landmarks, the MRI image is used to display the position of the TMS coil relative to the participant's brain in real time.

Follow-Up/Termination Procedures

Criteria for withdrawal will include worsening of current medical condition or development of a new medical condition, noncompliance with scheduling, testing or training procedures, and initiation of an exercise or rehabilitation program that could affect experimental results. Upon completion of all of the study sessions, participants will be encouraged to contact investigators if they have any questions, concerns, or comments regarding their participation in the study.

All procedures described are research procedures. No clinical care will be given. This protocol employs no medications or devices requiring IND/IDE. No radiation will be used in the study.

b. Study Volunteers

We plan to recruit 30 individuals with chronic (> 6 months) cortical or subcortical stroke affecting 1 hemisphere, with at least partial sparing of primary motor and premotor cortices. 15 nondisabled adults will also be included for comparison purposes for a total sample of 45. Inclusion and exclusion criteria are summarized in Table 1.

Table 1 (* = applies to stroke survivors only)

**Inclusion Criteria**
≥ 6 months post thromboembolic non-hemorrhagic hemispheric or hemorrhagic hemispheric lesion.*
Inability to actively extend the paretic wrist and fingers at least 20 degrees past neutral.*
**Exclusion Criteria**
Stroke < 6 months ago or affecting both hemispheres.*
Involvement of cerebellum, brainstem, or a large stroke with no sparing of primary motor or dorsal premotor cortices.*
History of craniotomy.
History of neurological disorder or disease (other than stroke).
Have had a seizure or have taken anti-seizure medications within the past 2 years.
History of orthopedic injury or disorder affecting shoulder or elbow function.

Less than 18 years of age.
Have a pacemaker, implanted pumps or stimulators, or metal objects inside the eye or skull.
Pregnancy.
Unable to perform the required movements.
Severe uncontrolled medical problems (e.g. cardiovascular disease, severe rheumatoid arthritis, arthritic joint deformity, active cancer or renal disease).
Serious cognitive deficits (defined as equivalent to a mini-mental state exam score of 24 or less) that would prevent their ability to give informed consent and/or perform the study tasks.

Recruitment

Stroke survivors will be recruited from the community by distributing flyers (attached) and via Internet announcements. Participants will also be recruited from the MedStar NRH Stroke Registry and from direct contact with health care providers. The MedStar NRH Stroke Registry database contains information about hundreds of stroke patients who were admitted to MedStar NRH and consented to be contacted in the future regarding rehabilitation-related studies for which they may be eligible. For those who are interested in participating, a phone interview will be performed using a phone script (attached) to screen the potential volunteer for suitability for the study and ensure the potential participant meets the specified inclusion criteria and does not have any exclusion criterion.

Healthy individuals will be recruited from the community by distributing flyers (attached), via Internet announcements and, for MedStar employees, via group email. Undue influence or coercion will be avoided with the recruitment of MedStar employees by sending the study announcement via email and allowing the volunteers to respond to the researchers if they are interested. In addition, the investigators will not attempt to recruit any MedStar employees whom they directly supervise.

c. Data Analysis

Using a 2-way repeated measures ANOVA with factors Cortical Site and Time, we expect a significant Interaction of Cortical Site x Time, such that performance improvement due to practice will be greater with pre-practice iTBS to PMd than to M1 or control sites.

We will also calculate the Pearson Product Moment Correlation Coefficient between pre-practice response time and change in response time with PMd iTBS + practice. We expect a significant correlation between slower baseline response times and larger response time improvements with PMd iTBS + practice.

Finally, again using a repeated measures ANOVA with factors Cortical Site and Time, we expect that, compared to baseline testing, the effects of PMd disruption will be greater after PMd iTBS + practice, indicating that it has taken on a more prominent role in affected arm reaching.

7. Risk/Benefits Assessment

a. Risks

Risk of anatomical MRI

Cautionary signs regarding the specific dangers of high magnetic fields are posted in the imaging facility. Participants will complete a comprehensive questionnaire to verify that they have no magnetic material on or inside their body or clothing before entering the scan room. A standard 1.5 Tesla magnet will be used. 1.5 Tesla scanners are used routinely for clinical evaluation of patients. We will follow the guidelines from the Bureau of Radiological Health, FDA to monitor the radio frequency deposition and time varying magnetic fields (dB/dt). Acoustic noise is generated in the magnet when the gradient coils are energized and de-energized in the magnetic fields to create MRI images. Participants will be required to wear earplugs during the scan. In general, this noise is not a significant problem in clinical scanners and no incident of hearing impairment has been reported. During the scan, patients will be able to communicate with investigators via an intercom system and will be given a call button that they can push if they want to stop the scan for any reason. This procedure is safe and should not produce any undue discomfort to the study volunteers. Some people may unexpectedly experience sensations of claustrophobia during the scan. All participants will be informed that they can push the call button at any time to immediately stop the scan and be brought out of the scanner. The main discomfort associated with the study is the need to remain quiet and still within the scanner for an extended period of time (10-15 minutes of actual scan time, plus a small amount of time to set-up the computer programs which collect the data).

Another possible risk is that of apparent abnormalities appearing unexpectedly on a participant's MRI. If study personnel have a concern regarding something that appears on the participant's MRI, Dr. Dromerick, the physician providing medical oversight for the study, will be consulted and will determine whether a neuroradiology consult is warranted. If so, one of Dr. Dromerick's colleagues in neuroradiology will determine whether the finding warrants further investigation. If so, the participant and if possible, their primary care physician will be contacted and informed of the finding. The decision as to whether to pursue additional testing lies solely with the participant and their physician. Because the scans are not optimized for clinical diagnostic purposes, they will not be made available for diagnostic purposes. This information is reflected in the consent form.

Risk of behavioral testing

There are minimal risks associated with the behavioral testing. Risks may include post-exercise muscle soreness, which is expected to resolve within 24 hours. This risk is minimized by offering frequent rest periods between bouts of reaching movements. Another potential risk is

the development of skin abrasions from repeated physical contact with the table or the target button. The risk of this occurring is minimal, though it may be slightly more likely in individuals who are taking anti-clotting medication. Any abrasion that occurs is expected to be mild and resolve within 1 week.

Risk of TMS

TMS became widely adopted in the late 1980s and early 1990s. Safety studies in human subjects reached encouraging conclusions, and TMS' widespread clinical and investigational use helped to establish a general consensus that it is safe in most subjects (Bridgers & Delaney 1989). Subsequent studies have confirmed the safety of single-pulse TMS in humans (Anand & Hotson 2002; Dodick et al 2010; Rossini & Rossi 2007; Wassermann 1998; 2000; Ziemann et al 1998), including children (Gilbert et al 2004).

Seizures have been reported using single-pulse TMS in three patients with cerebral infarcts, out of hundreds tested all over the world (Fauth et al 1992; Homberg & Netz 1989; Kandler 1990). The anatomical extent of these lesions has not been reported in all cases. There do not appear to be reports of seizures in patients with lesions that were completely subcortical. Only one case has been reported in which single-pulse TMS could produce seizures repeatedly in a single individual, who had a history of epilepsy (Classen et al 1995).

Individuals who sustain a severe stroke sometimes have seizure activity during the acute period post-stroke. To clarify this issue in terms of the eligibility of such individuals, we would like to set a required 2-year time period during which they were not taking any anti-epileptic drugs and did not have any seizure activity.

In regard to iTBS, Oberman et al (2011) performed an English language literature search, and reviewed 64 studies published from May 2004 to December 2009 in which cTBS and/or iTBS was applied. The majority of adverse events attributed to TBS was mild and occurred in only 5% of participants. The total sample size of participants was 1001 and the reported adverse events were (1) seizure in 1 healthy control subject during cTBS, (2) mild headache in 24 participants, (3) nonspecific discomfort in 5 patients with tinnitus, (4) mild discomfort due to cutaneous sensation and neck muscle contraction in 5 healthy control participants, (5) worsening tinnitus in 3 tinnitus patients, (6) nausea in 1 patient with Parkinson's Disease, (7) light-headedness or vagal responses in 11 healthy control participants, and (8) unilateral eye pain and lacrimation in 1 healthy control subject (which ceased upon cessation of the treatment session). Subsequent studies have continued to support the safety and tolerability of TBS, including many studies in stroke patients (Ackerley et al 2013; Di Lazzaro et al 2013; Hsu et al 2013; Kindler et al 2012; Koch et al 2012; Szaflarski et al 2011; Talelli et al 2012), and even in children (Wu et al 2012).

The one incident of seizure induced by TBS (Oberman & Pascual-Leone 2009) occurred in a 33 year old healthy man with no risk factors for epilepsy. The seizure occurred following approximately 50 trains (10 seconds) of cTBS to the primary motor cortex at an intensity of 100% of resting motor threshold (RMT). cTBS (continuous theta burst stimulation) is another type of TBS paradigm where 200 trains (sometimes 100 trains) of stimuli are delivered

continuously. We will not use cTBS for this study and, for our iTBS paradigm, we plan to deliver stimuli at 80% - 90% of active motor threshold (AMT), which is nearly always a significantly lower intensity than the RMT.

The most common side-effect induced by single-pulse TMS or TBS (Bae et al 2007; Oberman et al 2011; Ragert et al 2009; Rossi et al 2009; Rothkegel et al 2009) is mild headache due to the brief scalp muscle twitches which can occur with each stimulus. This discomfort is usually mild, described as a "tension headache" sensation and resolves completely within 24 hours.

b. Benefits

There will be no direct benefit from participating in the study. However, we expect the study results to contribute to the development of more effective rehabilitation interventions for arm impairment after stroke and other neurological disorders and injuries.

8. Reporting of Serious or Unexpected Adverse Events and Unanticipated Problems

We do not anticipate that participation in this study will cause the participant any injury, illness and/or exacerbation of preexisting conditions. In the unlikely event that an unexpected or serious adverse event does occur, we will immediately report them to MedStar Health Research Institute by completing a MHRI Serious Adverse Event Report form. The principal investigator will report the adverse event to the IRB within 24 - 48 hours of being notified of the event. The report will be addressed to MedStar Health Research Institute, Office of Research Integrity, 6525 Belcrest Rd., Suite 700, Hyattsville, Maryland 20782.

9. Disposition of Data

A computer file linking the participants' personal information to numerical participant IDs will be kept in a password-protected file on a password-protected computer accessed only by study investigators and immediate staff. Hard copies of data will be stored in locked file cabinets. Consent forms with personally identifiable data will be stored separately from study data in a locked file cabinet. Data will be entered into the database by the investigators only. All electronic data will be stored according to a coded participant ID. Computers on which data are analyzed will be password protected. Data will be stored in archive format following completion of the study, using appropriate security procedures. Data will be kept for 6 years after which the principal investigator will be responsible for destruction of data. Computer files will be deleted and hard copies will be shredded using shredding services hired by the hospital.

10. Modification of Protocol

The MHRI IRB will be notified by letter 30 days prior to any modification to the protocol and/or consent form. Any deviation from the protocol that may have an effect on the safety or rights of the participant or the integrity of the study will be reported as soon as the deviation is identified.

Major modifications to the research protocol and any modifications that could potentially increase risk to participants will be submitted for approval prior to implementation. All other amendments will be submitted with the annual continuing review report for acceptance.

11. Departure from the Protocol

There will be no departure from the approved protocol unless it is first approved by the MHRI IRB.

12. Roles and Responsibilities of Study Personnel

The principal investigator, Dr. Michelle Harris-Love will oversee all aspects of this project, including determining potential participant eligibility, training study personnel in equipment setup and collection of physiological and behavioral data, and performing data reduction, analysis, interpretation and dissemination. Dr. Alexander Dromerick, a stroke neurologist with extensive experience in both rehabilitation research and post-stroke care, will perform the neurological exam and provide medical oversight for the study. He will verify that the participant meets all of the eligibility criteria, classify the type of stroke according to clinically accepted criteria, and provide consultation should any questions arise regarding the participant's medical status. When Dr. Dromerick is not available, Dr. Peter Turkeltaub will perform the neurological exam instead. In addition, as stated above, Dr. Dromerick will consult with a Washington Hospital Center neuroradiologist should any apparent abnormalities appear on a participant's MRI. Evan Chan and Rachael Harrington will assist with screening and enrollment, perform equipment setup, data collection and data analysis.

13. Medical Care for Research-related Injuries

Great care will be taken to prevent research-related injuries and the risk of a research-related injury or illness for this study is very low. In the unlikely event of a research-related injury or illness, or any non-research-related injury or illness occurring during the time the participant is enrolled in the study, the participant's medical insurance or other third-party payer is expected to provide coverage for any necessary treatment. This is clearly stated in the consent form.

14. Signature of Principal Investigator

I have read the foregoing protocol and agree to conduct the study as outlined herein.

Date: 8/17/17

Michelle Harris-Love, PT, PhD

References

Ackerley SJ, Stinear CM, Barber PA, Byblow WD. 2010. Combining theta burst stimulation with training after subcortical stroke. *Stroke* 41:1568-72

Ackerley SJ, Stinear CM, Barber PA, Byblow WD. 2013. Priming sensorimotor cortex to enhance task-specific training after subcortical stroke. *Clin Neurophysiol*

Anand S, Hotson J. 2002. Transcranial magnetic stimulation: neurophysiological applications and safety. *Brain Cogn* 50:366-86

Bae EH, Schrader LM, Machii K, Alonso-Alonso M, Riviello JJ, Jr., et al. 2007. Safety and tolerability of repetitive transcranial magnetic stimulation in patients with epilepsy: a review of the literature. *Epilepsy Behav* 10:521-8

Bridgers SL, Delaney RC. 1989. Transcranial magnetic stimulation: an assessment of cognitive and other cerebral effects. *Neurology* 39:417-9

Broeks JG, Lankhorst GJ, Rumping K, Prevo AJ. 1999. The long-term outcome of arm function after stroke: results of a follow-up study. *Disabil Rehabil* 21:357-64

Burke MW, Kupers R, Ptito M. 2012. Adaptive neuroplastic responses in early and late hemispherectomized monkeys. *Neural Plast* 2012:852423

Choi JT, Vining EP, Mori S, Bastian AJ. 2010. Sensorimotor function and sensorimotor tracts after hemispherectomy. *Neuropsychologia* 48:1192-9

Classen J, Witte OW, Schlaug G, Seitz RJ, Holthausen H, Benecke R. 1995. Epileptic seizures triggered directly by focal transcranial magnetic stimulation. *Electroencephalogr Clin Neurophysiol* 94:19-25

Desrosiers J, Noreau L, Rochette A, Bourbonnais D, Bravo G, Bourget A. 2006. Predictors of long-term participation after stroke. *Disabil Rehabil* 28:221-30

Di Lazzaro V, Pilato F, Dileone M, Profice P, Capone F, et al. 2008. Modulating cortical excitability in acute stroke: a repetitive TMS study. *Clin Neurophysiol* 119:715-23

Di Lazzaro V, Profice P, Pilato F, Capone F, Ranieri F, et al. 2010. Motor cortex plasticity predicts recovery in acute stroke. *Cereb Cortex* 20:1523-8

Di Lazzaro V, Rothwell JC, Talelli P, Capone F, Ranieri F, et al. 2013. Inhibitory theta burst stimulation of affected hemisphere in chronic stroke: a proof of principle, sham-controlled study. *Neurosci Lett* 553:148-52

Dodick DW, Schembri CT, Helmuth M, Aurora SK. 2010. Transcranial magnetic stimulation for migraine: a safety review. *Headache* 50:1153-63

Fauth C, Meyer BU, Prosiegel M, Zihl J, Conrad B. 1992. Seizure induction and magnetic brain stimulation after stroke. *Lancet* 339:362

Gilbert DL, Garvey MA, Bansal AS, Lipps T, Zhang J, Wassermann EM. 2004. Should transcranial magnetic stimulation research in children be considered minimal risk? *Clin Neurophysiol* 115:1730-9

Homberg V, Netz J. 1989. Generalised seizures induced by transcranial magnetic stimulation of motor cortex. *Lancet* 2:1223

Honda N, Matuoka T, Sawada Y, Nakano N, Suwen L, et al. 2010. Reorganization of sensorimotor function after functional hemispherectomy studied using near-infrared spectroscopy. *Pediatr Neurosurg* 46:313-7

Hsu YF, Huang YZ, Lin YY, Tang CW, Liao KK, et al. 2013. Intermittent theta burst stimulation over ipsilesional primary motor cortex of subacute ischemic stroke patients: a pilot study. *Brain Stimul* 6:166-74

Johansen-Berg H, Rushworth MF, Bogdanovic MD, Kischka U, Wimalaratna S, Matthews PM. 2002. The role of ipsilateral premotor cortex in hand movement after stroke. *Proc Natl Acad Sci U S A* 99:14518-23

Kandler R. 1990. Safety of transcranial magnetic stimulation. *Lancet* 335:469-70

Kindler J, Schumacher R, Cazzoli D, Gutbrod K, Koenig M, et al. 2012. Theta burst stimulation over the right Broca's homologue induces improvement of naming in aphasic patients. *Stroke* 43:2175-9
Koch G, Bonni S, Giacobbe V, Bucchi G, Basile B, et al. 2012. theta-burst stimulation of the left hemisphere accelerates recovery of hemispatial neglect. *Neurology* 78:24-30
Likhi M, Jidesh VV, Kanagaraj R, George JK. 2013. Does trunk, arm, or leg control correlate best with overall function in stroke subjects? *Top Stroke Rehabil* 20:62-7
Lozano R, Naghavi M, Foreman K, Lim S, Shibuya K, et al. 2012. Global and regional mortality from 235 causes of death for 20 age groups in 1990 and 2010: a systematic analysis for the Global Burden of Disease Study 2010. *Lancet* 380:2095-128
Nowak DA, Grefkes C, Ameli M, Fink GR. 2009. Interhemispheric competition after stroke: brain stimulation to enhance recovery of function of the affected hand. *Neurorehabil Neural Repair* 23:641-56
Oberman L, Edwards D, Eldaief M, Pascual-Leone A. 2011. Safety of theta burst transcranial magnetic stimulation: a systematic review of the literature. *J Clin Neurophysiol* 28:67-74
Oberman LM, Pascual-Leone A. 2009. Report of seizure induced by continuous theta burst stimulation. *Brain Stimul* 2:246-7
Ovbiagele B, Goldstein LB, Higashida RT, Howard VJ, Johnston SC, et al. 2013. Forecasting the Future of Stroke in the United States: A Policy Statement From the American Heart Association and American Stroke Association. *Stroke*
Ragert P, Camus M, Vandermeeren Y, Dimyan MA, Cohen LG. 2009. Modulation of effects of intermittent theta burst stimulation applied over primary motor cortex (M1) by conditioning stimulation of the opposite M1. *J Neurophysiol* 102:766-73
Rossi S, Hallett M, Rossini PM, Pascual-Leone A. 2009. Safety, ethical considerations, and application guidelines for the use of transcranial magnetic stimulation in clinical practice and research. *Clin Neurophysiol* 120:2008-39
Rossini PM, Rossi S. 2007. Transcranial magnetic stimulation: diagnostic, therapeutic, and research potential. *Neurology* 68:484-8
Rothkegel H, Sommer M, Rammsayer T, Trenkwalder C, Paulus W. 2009. Training effects outweigh effects of single-session conventional rTMS and theta burst stimulation in PD patients. *Neurorehabil Neural Repair* 23:373-81
Szaflarski JP, Vannest J, Wu SW, DiFrancesco MW, Banks C, Gilbert DL. 2011. Excitatory repetitive transcranial magnetic stimulation induces improvements in chronic post-stroke aphasia. *Med Sci Monit* 17:CR132-9
Talelli P, Greenwood RJ, Rothwell JC. 2007. Exploring Theta Burst Stimulation as an intervention to improve motor recovery in chronic stroke. *Clin Neurophysiol* 118:333-42
Talelli P, Wallace A, Dileone M, Hoad D, Cheeran B, et al. 2012. Theta burst stimulation in the rehabilitation of the upper limb: a semirandomized, placebo-controlled trial in chronic stroke patients. *Neurorehabil Neural Repair* 26:976-87
Ward N. 2011. Assessment of cortical reorganisation for hand function after stroke. *J Physiol* 589:5625-32
Ward NS, Cohen LG. 2004. Mechanisms underlying recovery of motor function after stroke. *Arch Neurol* 61:1844-8
Wassermann EM. 1998. Risk and safety of repetitive transcranial magnetic stimulation: report and suggested guidelines from the International Workshop on the Safety of Repetitive Transcranial Magnetic Stimulation, June 5-7, 1996. *Electroencephalogr Clin Neurophysiol* 108:1-16
Wassermann EM. 2000. Side effects of repetitive transcranial magnetic stimulation. *Depress Anxiety* 12:124-9

Wolf SL, Winstein CJ, Miller JP, Thompson PA, Taub E, et al. 2008. Retention of upper limb function in stroke survivors who have received constraint-induced movement therapy: the EXCITE randomised trial. *Lancet Neurol* 7:33-40

Wu SW, Shahana N, Huddleston DA, Lewis AN, Gilbert DL. 2012. Safety and tolerability of theta-burst transcranial magnetic stimulation in children. *Dev Med Child Neurol* 54:636-9

Ziemann U, Steinhoff BJ, Tergau F, Paulus W. 1998. Transcranial magnetic stimulation: its current role in epilepsy research. *Epilepsy Res* 30:11-30
