# Supplementary material for: Upper extremity kinematics: development of a quantitative measure of impairment severity and dissimilarity after stroke
Source: PeerJ. 2023 Dec 8;11:e16374. doi: 10.7717/peerj.16374 (PMC10712307; doi:10.7717/peerj.16374)
Supplement: Supplemental Information 2 [file peerj-11-16374-s002.docx]

**TBS Study Test Sheet (05/02/2018)**

*****Turn on power strip on the back wall. *****

**In-person Screening (Reaching Test for average reaction time)**

Paretic/ side: L / R

1. Reaching set-up measures

Maximum reach ____ cm 80% max reach ____ cm

1. Passive ideal hand path NDI file name: L button ____ R button ____ (Non Paretic)

L button ____ R button ____ (Paretic)

1. Unilateral **Non** **Paretic** SRT Reaching Test (10 trials x 2 blocks: 80% MAX Reach, **reach in closed fist**)

NDI file names: ________________ E-prime file name: ____________ (SimpleRT_eval)

**Reach Screening Test**

1. Unilateral **Paretic**  SRT Reaching Test (10 trials x 2 blocks: 80% MAX Reach, **reach in closed fist**)

NDI file names: ________________ E-prime file name: ____________ (SimpleRT_eval)

Mean: ­­­_____________

SD: _______________

SD: ______________


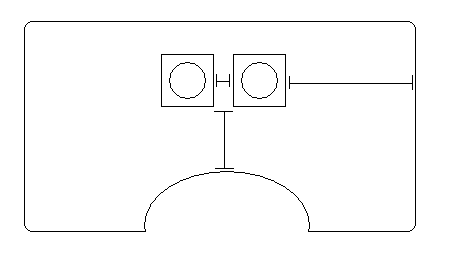


b/w button: _1.3_ cm

C: _51_ cm

D: 80% MAX = ____ cm

E: ____ cm

F: ____ cm

C

D


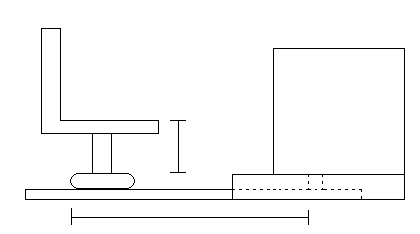


F

E

**Adverse Events: Y / N (if yes, explain ___________________________________________________________________)**

**Comments: ________________________________________________________________________________________**

**______________________________________________________________________________________________________________________________________________________________________________________________________________________________________________________________________________________________________**

**TMS Target Determination**

1. Contralesional M1 for both BB/TB & FDI ____

**(determine target that elicits the largest and most consistent MEPs from the specified muscles, may use muscle activation to determine best site)**

1. Ipsilesional M1 for both BB/TB & FDI ____

**(determine target that elicits the largest and most consistent MEPs from the specified muscles, if no MEPs elicited, then mirror the contralesional side)**

1. MEPs from affected FDI – Single-Pulse at Maximum Stimulator Output over hotspot for M1 for FDI x10 > 50 uV = ___/10

**(determine if MEPs are present at rest) Save File. File Name__________**

1. Contralesional PMd ____

**Point between the middle and posterior 2/3 of superior frontal sulcus**

1. Ipsilesional PMd ____

**Point between the middle and posterior 2/3 of superior frontal sulcus**

1. Contralesional DLPFC ____

**Anterior 2/3 of superior frontal sulcus**

1. Contralesional ___ (muscle) RMT = ____% (5/10 trials have > 50 uV)
2. Ipsilesional ___ (muscle) RMT = ____% (5/10 trials have > 50 uV)
3. Spreading test: Contralesional PMd **(<= 1/10 trials with MEPs) = ___% RMT**
4. Spreading test: Ipsilesional PMd **(<= 1/10 trials with MEPs) = ___% RMT**
5. **Randomization table entry for sites 1, 2, and 3 ______________________________**

Note:

1. **Attach electrodes for both arms for this and subsequent sessions.**
2. For healthy subjects, use hemisphere Contralateral to dominant arm as if **Contralesional** hemisphere, use hemisphere Ipsilateral to dominant arm as if **Ipsilesional** hemisphere
3. PMd is defined as the center of the **posterior third** of **middle frontal gyrus** (F2), between the superior and inferior frontal sulci and just anterior to the precentral sulcus and motor hotspot (Adhab et al 2010).

**Adverse Events: Y / N (if yes, explain ___________________________________________________________________)**

**Comments: ______________________________________________________________________________________________________________________________________________________________________________________________________________________________________________________________________________________________________**

**Target (#, not specific site) ______ Day 1 – TMS Disruption**

1) Contralesional ___ (muscle) RMT = ____% (5/10 trials have > 50 uV)

___% RMT= ___ % for DP disruption, ISI = 25 ms

2) Contralesional ___ (muscle) AMT = ____% (5/10 trials have > 100 uV)

EMG activation ___ to ___ RMS uV

3) Passive ideal hand path NDI file name:

L button ____ R button ____ (Paretic)

4) Instruct patients: “When one of the target lights comes on, quickly reach out to touch that target and then return to the start position.”

5) Unilateral Paretic CRT Reaching Test with disruption (80% MAX Reach, **reach in closed fist**)

10 blocks of 10; 3 w/o TMS, 6 w/ TMS (150, 200, 250 ms), 1 catch (ChoiceRT_Paired Pulse)

| E-Prime | NDI | Block | Rest (Y/N) | Circle bad trials | | | | | | | | | | | |
| --- | --- | --- | --- | --- | --- | --- | --- | --- | --- | --- | --- | --- | --- | --- | --- |
|  |  | **1** |  | 1 | 2 | 3 | 4 | 5 | 6 | 7 | 8 | 9 | 10 |  |  |
|  |  | **2** |  | 11 | 12 | 13 | 14 | 15 | 16 | 17 | 18 | 19 | 20 |  |  |
|  |  | **3** |  | 21 | 22 | 23 | 24 | 25 | 26 | 27 | 28 | 29 | 30 |  |  |
|  |  | **4** |  | 31 | 32 | 33 | 34 | 35 | 36 | 37 | 38 | 39 | 40 |  |  |
|  |  | **5** |  | 41 | 42 | 43 | 44 | 45 | 46 | 47 | 48 | 49 | 50 |  |  |
|  |  | **6** |  | 51 | 52 | 53 | 54 | 55 | 56 | 57 | 58 | 59 | 60 |  |  |
|  |  | **7** |  | 61 | 62 | 63 | 64 | 65 | 66 | 67 | 68 | 69 | 70 |  |  |
|  |  | **8** |  | 71 | 72 | 73 | 74 | 75 | 76 | 77 | 78 | 79 | 80 |  |  |
|  |  | **9** |  | 81 | 82 | 83 | 84 | 85 | 86 | 87 | 88 | 89 | 90 |  |  |
|  |  | **10** |  | 91 | 92 | 93 | 94 | 95 | 96 | 97 | 98 | 99 | 100 |  |  |

**Adverse Events: Y / N (if yes, explain ___________________________________________________________________)**

**Comments: ________________________________________________________________________________________**

**____________________________________________________________________________________________________________________________________________________________________________________________________**

IF changed:

MAX reach: ____ cm

b/w button: ____ cm

C: ____ cm

D: 60% MAX = ____ cm

80% MAX = ____ cm

100% MAX = ____ cm

E: ____ cm

F: ____ cm

**Target (#, not specific site) ______ Day 2 – Reaching + TBS**

1) Contralesional ___ (muscle) AMT = ____% (5/10 trials have > 100 uV)

EMG activation ___ to ___ RMS uV; ___% AMT= ___ % for TBS

2) Passive ideal hand path NDI file name:

One button: 80% MAX Reach: ____ (Paretic)

**Reach Pre-Test**

3) Unilateral Paretic SRT Reaching Test (10 trials x 3 blocks: **reach in closed fist**) (SimpleRT_Test)

60% MAX Reach: NDI file names: ________________ E-prime file name: ______________

80% MAX Reach: NDI file names: ________________ E-prime file name: ______________

100% MAX Reach: NDI file names: ________________ E-prime file name: ______________

4) Intermittent TBS (10 bursts x 20 trains; 3.3 minutes) ________

5) Unilateral Paretic SRT Reaching **Practice** (10 trials x 20 blocks: **reach in closed fist**) (SimpleRT_Train)

80% MAX Reach: NDI file names (first 2 blocks only): ______________ E-prime file name: ____________

6) Break _10 minutes

7) Unilateral Paretic SRT Reaching Test (10 trials x 2 blocks: **reach in closed fist**) (SimpleRT_Test)

80% MAX Reach: NDI file names: ________________ E-prime file name: ____________

**Adverse Events: Y / N (if yes, explain ___________________________________________________________________)**

**Comments: ________________________________________________________________________________________**

**____________________________________________________________________________________________________________________________________________________________________________________________________**

**Target (#, not specific site) ______ Day 3 – Reaching + TMS Disruption**

IF changed:

MAX reach: ____ cm

b/w button: ____ cm

C: ____ cm

D: 60% MAX = ____ cm

80% MAX = ____ cm

100% MAX = ____ cm

E: ____ cm

F: ____ cm

1) Contralesional ___ (muscle) RMT = ____% (5/10 trials have > 50 uV)

___% RMT= ___ % for DP disruption, ISI = 25 ms

2) Passive ideal hand path NDI file name:

One button: 60% MAX Reach: ____ (Paretic)

One button: 80% MAX Reach: ____ (Paretic)

One button: 100% MAX Reach: ____ (Paretic)

L button ____ R button ____ (Paretic)

**Reach 24-Hr Post-Test**

3) Unilateral Paretic SRT Reaching Test (10 trials x 3 blocks: **reach in closed fist**)

60% MAX Reach: NDI file names: ______________ E-prime file name: ____________

80% MAX Reach: NDI file names: ______________ E-prime file name: ____________

100% MAX Reach: NDI file names: _____________ E-prime file name: ____________

4) Instruct patients: “When one of the target lights comes on, quickly reach out to touch that target and then return to the start position.”

**Reach + TMS Disruption (Site 1)**

5) Unilateral Paretic CRT Reaching Test with disruption (80% MAX Reach, **reach in closed fist**)

10 blocks of 10; 3 w/o TMS, 6 w/ TMS (150, 200, 250 ms), 1 catch (ChoiceRT_Paired Pulse)

| E-Prime | NDI | Block | Rest (Y/N) | Circle bad trials | | | | | | | | | | | |
| --- | --- | --- | --- | --- | --- | --- | --- | --- | --- | --- | --- | --- | --- | --- | --- |
|  |  | **1** |  | 1 | 2 | 3 | 4 | 5 | 6 | 7 | 8 | 9 | 10 |  |  |
|  |  | **2** |  | 11 | 12 | 13 | 14 | 15 | 16 | 17 | 18 | 19 | 20 |  |  |
|  |  | **3** |  | 21 | 22 | 23 | 24 | 25 | 26 | 27 | 28 | 29 | 30 |  |  |
|  |  | **4** |  | 31 | 32 | 33 | 34 | 35 | 36 | 37 | 38 | 39 | 40 |  |  |
|  |  | **5** |  | 41 | 42 | 43 | 44 | 45 | 46 | 47 | 48 | 49 | 50 |  |  |
|  |  | **6** |  | 51 | 52 | 53 | 54 | 55 | 56 | 57 | 58 | 59 | 60 |  |  |
|  |  | **7** |  | 61 | 62 | 63 | 64 | 65 | 66 | 67 | 68 | 69 | 70 |  |  |
|  |  | **8** |  | 71 | 72 | 73 | 74 | 75 | 76 | 77 | 78 | 79 | 80 |  |  |
|  |  | **9** |  | 81 | 82 | 83 | 84 | 85 | 86 | 87 | 88 | 89 | 90 |  |  |
|  |  | **10** |  | 91 | 92 | 93 | 94 | 95 | 96 | 97 | 98 | 99 | 100 |  |  |

**Adverse Events: Y / N (if yes, explain ___________________________________________________________________)**

**Comments: ________________________________________________________________________________________**

**____________________________________________________________________________________________________________________________________________________________________________________________________**

**Target (#, not specific site) ______ Day 1 – TMS Disruption**

Mean: ­­­_____________

SD: _______________

*Compare Mean and SD to page 1*

SD: ______________

1) Passive ideal hand path NDI file name:

L button ____ R button ____ (Paretic)

**Reach “Washout” Test**

2) Unilateral **Paretic**  SRT Reaching Test (10 trials x 2 blocks: 80% MAX Reach, **reach in closed fist**)

NDI file names: __________________ E-prime file name: ____________ (SimpleRT_eval)

3) Contralesional ___ (muscle) RMT = ____% (5/10 trials have > 50 uV)

___% RMT= ___ % for DP disruption, ISI = 25 ms

4) Contralesional ___ (muscle) AMT = ____% (5/10 trials have > 100 uV)

EMG activation ___ to ___ RMS uV

5) Instruct patients: “When one of the target lights comes on, quickly reach out to touch that target and then return to the start position.”

6) Unilateral Paretic CRT Reaching Test with disruption (Site 2) (80% MAX Reach, **reach in closed fist**)

10 blocks of 10; 3 w/o TMS, 6 w/ TMS (150, 200, 250 ms), 1 catch (ChoiceRT_Paired Pulse)

| E-Prime | NDI | Block | Rest (Y/N) | Circle bad trials | | | | | | | | | | | |
| --- | --- | --- | --- | --- | --- | --- | --- | --- | --- | --- | --- | --- | --- | --- | --- |
|  |  | **1** |  | 1 | 2 | 3 | 4 | 5 | 6 | 7 | 8 | 9 | 10 |  |  |
|  |  | **2** |  | 11 | 12 | 13 | 14 | 15 | 16 | 17 | 18 | 19 | 20 |  |  |
|  |  | **3** |  | 21 | 22 | 23 | 24 | 25 | 26 | 27 | 28 | 29 | 30 |  |  |
|  |  | **4** |  | 31 | 32 | 33 | 34 | 35 | 36 | 37 | 38 | 39 | 40 |  |  |
|  |  | **5** |  | 41 | 42 | 43 | 44 | 45 | 46 | 47 | 48 | 49 | 50 |  |  |
|  |  | **6** |  | 51 | 52 | 53 | 54 | 55 | 56 | 57 | 58 | 59 | 60 |  |  |
|  |  | **7** |  | 61 | 62 | 63 | 64 | 65 | 66 | 67 | 68 | 69 | 70 |  |  |
|  |  | **8** |  | 71 | 72 | 73 | 74 | 75 | 76 | 77 | 78 | 79 | 80 |  |  |
|  |  | **9** |  | 81 | 82 | 83 | 84 | 85 | 86 | 87 | 88 | 89 | 90 |  |  |
|  |  | **10** |  | 91 | 92 | 93 | 94 | 95 | 96 | 97 | 98 | 99 | 100 |  |  |

**Adverse Events: Y / N (if yes, explain ___________________________________________________________________)**

**Comments: ________________________________________________________________________________________**

**____________________________________________________________________________________________________________________________________________________________________________________________________**

IF changed:

MAX reach: ____ cm

b/w button: ____ cm

C: ____ cm

D: 60% MAX = ____ cm

80% MAX = ____ cm

100% MAX = ____ cm

E: ____ cm

F: ____ cm

**Target (#, not specific site) ______ Day 2 – Reaching + TBS**

1) Contralesional ___ (muscle) AMT = ____% (5/10 trials have > 100 uV)

EMG activation ___ to ___ RMS uV; ___% AMT= ___ % for TBS

2) Passive ideal hand path NDI file name:

One button: 80% MAX Reach: ____ (Paretic)

**Reach Pre-Test**

3) Unilateral Paretic SRT Reaching Test (10 trials x 3 blocks: **reach in closed fist**) (SimpleRT_Test)

60% MAX Reach: NDI file names: ______________ E-prime file name: ____________

80% MAX Reach: NDI file names: ______________ E-prime file name: ____________

100% MAX Reach: NDI file names: _____________ E-prime file name: ____________

4) Intermittent TBS (10 bursts x 20 trains; 3.3 minutes) ________

**Reach Practice**

5) Unilateral Paretic SRT Reaching Practice (10 trials x 20 blocks: **reach in closed fist**) (SimpleRT_Train)

80% MAX Reach: **NDI file names (first 2 blocks only):** _______________ E-prime file name: ____________

6) Break _10 minutes _

**Reach 10-minutes Post-Test**

7) Unilateral Paretic SRT Reaching Test (10 trials x 3 blocks: **reach in closed fist**) (SimpleRT_Test)

80% MAX Reach: NDI file names: ________________ E-prime file name: ____________

**Adverse Events: Y / N (if yes, explain ___________________________________________________________________)**

**Comments: ________________________________________________________________________________________**

**____________________________________________________________________________________________________________________________________________________________________________________________________**

**Target (#, not specific site) ______ Day 3 – Reaching + TMS Disruption**

IF changed:

MAX reach: ____ cm

b/w button: ____ cm

C: ____ cm

D: 60% MAX = ____ cm

80% MAX = ____ cm

100% MAX = ____ cm

E: ____ cm

F: ____ cm

1) Contralesional ___ (muscle) RMT = ____% (5/10 trials have > 50 uV)

___% RMT= ___ % for DP disruption, ISI = 25 ms

2) Passive ideal hand path NDI file name:

One button: 60% MAX Reach: ____ (Paretic)

One button: 80% MAX Reach: ____ (Paretic)

One button: 100% MAX Reach: ____ (Paretic)

L button ____ R button ____ (Paretic)

3) Unilateral Paretic SRT Reaching Test (10 trials x 3 blocks: **reach in closed fist**)

60% MAX Reach: NDI file names: ______________ E-prime file name: ____________

80% MAX Reach: NDI file names: ______________ E-prime file name: ____________

100% MAX Reach: NDI file names: _____________ E-prime file name: ____________

4) Instruct patients: “When one of the target lights comes on, quickly reach out to touch that target and then return to the start position.”

5) Unilateral Paretic CRT Reaching Test with disruption (80% MAX Reach, **reach in closed fist**)

10 blocks of 10; 3 w/o TMS, 6 w/ TMS (150, 200, 250 ms), 1 catch (ChoiceRT_Paired Pulse)

| E-Prime | NDI | Block | Rest (Y/N) | Circle bad trials | | | | | | | | | | | |
| --- | --- | --- | --- | --- | --- | --- | --- | --- | --- | --- | --- | --- | --- | --- | --- |
|  |  | **1** |  | 1 | 2 | 3 | 4 | 5 | 6 | 7 | 8 | 9 | 10 |  |  |
|  |  | **2** |  | 11 | 12 | 13 | 14 | 15 | 16 | 17 | 18 | 19 | 20 |  |  |
|  |  | **3** |  | 21 | 22 | 23 | 24 | 25 | 26 | 27 | 28 | 29 | 30 |  |  |
|  |  | **4** |  | 31 | 32 | 33 | 34 | 35 | 36 | 37 | 38 | 39 | 40 |  |  |
|  |  | **5** |  | 41 | 42 | 43 | 44 | 45 | 46 | 47 | 48 | 49 | 50 |  |  |
|  |  | **6** |  | 51 | 52 | 53 | 54 | 55 | 56 | 57 | 58 | 59 | 60 |  |  |
|  |  | **7** |  | 61 | 62 | 63 | 64 | 65 | 66 | 67 | 68 | 69 | 70 |  |  |
|  |  | **8** |  | 71 | 72 | 73 | 74 | 75 | 76 | 77 | 78 | 79 | 80 |  |  |
|  |  | **9** |  | 81 | 82 | 83 | 84 | 85 | 86 | 87 | 88 | 89 | 90 |  |  |
|  |  | **10** |  | 91 | 92 | 93 | 94 | 95 | 96 | 97 | 98 | 99 | 100 |  |  |

**Adverse Events: Y / N (if yes, explain ___________________________________________________________________)**

**Comments: ________________________________________________________________________________________**

Mean: ­­­_____________

SD: _______________

*Compare Mean and SD to page 6*

SD: ______________

**Target (#, not specific site) ______ Day 1 – TMS Disruption**

1) Passive ideal hand path NDI file name:

L button ____ R button ____ (Paretic)

**Reach “Washout” Test**

2) Unilateral **Paretic** SRT Reaching Test (10 trials x 2 blocks: 80% MAX Reach, **reach in closed fist**)

NDI file names: __________________ E-prime file name: ____________ (SimpleRT_eval)

3) Contralesional ___ (muscle) RMT = ____% (5/10 trials have > 50 uV)

___% RMT= ___ % for DP disruption, ISI = 25 ms

4) Contralesional ___ (muscle) AMT = ____% (5/10 trials have > 100 uV)

EMG activation ___ to ___ RMS uV

5) Instruct patients: “When one of the target lights comes on, quickly reach out to touch that target and then return to the start position.”

6) Unilateral Paretic CRT Reaching Test with disruption (80% MAX Reach, **reach in closed fist**)

10 blocks of 10; 3 w/o TMS, 6 w/ TMS (150, 200, 250 ms), 1 catch (ChoiceRT_Paired Pulse)

| E-Prime | NDI | Block | Rest (Y/N) | Circle bad trials | | | | | | | | | | | |
| --- | --- | --- | --- | --- | --- | --- | --- | --- | --- | --- | --- | --- | --- | --- | --- |
|  |  | **1** |  | 1 | 2 | 3 | 4 | 5 | 6 | 7 | 8 | 9 | 10 |  |  |
|  |  | **2** |  | 11 | 12 | 13 | 14 | 15 | 16 | 17 | 18 | 19 | 20 |  |  |
|  |  | **3** |  | 21 | 22 | 23 | 24 | 25 | 26 | 27 | 28 | 29 | 30 |  |  |
|  |  | **4** |  | 31 | 32 | 33 | 34 | 35 | 36 | 37 | 38 | 39 | 40 |  |  |
|  |  | **5** |  | 41 | 42 | 43 | 44 | 45 | 46 | 47 | 48 | 49 | 50 |  |  |
|  |  | **6** |  | 51 | 52 | 53 | 54 | 55 | 56 | 57 | 58 | 59 | 60 |  |  |
|  |  | **7** |  | 61 | 62 | 63 | 64 | 65 | 66 | 67 | 68 | 69 | 70 |  |  |
|  |  | **8** |  | 71 | 72 | 73 | 74 | 75 | 76 | 77 | 78 | 79 | 80 |  |  |
|  |  | **9** |  | 81 | 82 | 83 | 84 | 85 | 86 | 87 | 88 | 89 | 90 |  |  |
|  |  | **10** |  | 91 | 92 | 93 | 94 | 95 | 96 | 97 | 98 | 99 | 100 |  |  |

**Adverse Events: Y / N (if yes, explain ___________________________________________________________________)**

**Comments: ________________________________________________________________________________________**

**____________________________________________________________________________________________________________________________________________________________________________________________________**

IF changed:

MAX reach: ____ cm

b/w button: ____ cm

C: ____ cm

D: 60% MAX = ____ cm

80% MAX = ____ cm

100% MAX = ____ cm

E: ____ cm

F: ____ cm

**Target (#, not specific site) ______ Day 2 – Reaching + TBS**

1) Contralesional ___ (muscle) AMT = ____% (5/10 trials have > 100 uV)

EMG activation ___ to ___ RMS uV; ___% AMT= ___ % for TBS

2) Passive ideal hand path NDI file name:

One button: 80% MAX Reach: ____ (Paretic)

3) Unilateral Paretic / SRT Reaching Test (10 trials x 3 blocks: **reach in closed fist**) (SimpleRT_Test)

60% MAX Reach: NDI file names: _______________ E-prime file name: ____________

80% MAX Reach: NDI file names: _______________ E-prime file name: ____________

100% MAX Reach: NDI file names: ______________ E-prime file name: ____________

4) Intermittent TBS (10 bursts x 20 trains; 3.3 minutes) ________

5) Unilateral Paretic SRT Reaching **Practice** (10 trials x 20 blocks: **reach in closed fist**) (SimpleRT_Train)

80% MAX Reach: **NDI file names (first 2 blocks only)**: ______________ E-prime file name: ____________

6) Break _10 minutes__

7) Unilateral Paretic SRT Reaching Test (10 trials x 2 blocks: **reach in closed fist**) (SimpleRT_Test)

80% MAX Reach: NDI file names: __________________ E-prime file name: ____________

**Adverse Events: Y / N (if yes, explain ___________________________________________________________________)**

**Comments: ________________________________________________________________________________________**

**____________________________________________________________________________________________________________________________________________________________________________________________________**

**Target (#, not specific site) ______ Day 3 – Reaching + TMS Disruption**

IF changed:

MAX reach: ____ cm

b/w button: ____ cm

C: ____ cm

D: 60% MAX = ____ cm

80% MAX = ____ cm

100% MAX = ____ cm

E: ____ cm

F: ____ cm

1) Contralesional ___ (muscle) RMT = ____% (5/10 trials have > 50 uV)

___% RMT= ___ % for DP disruption, ISI = 25 ms

2) Passive ideal hand path NDI file name:

One button: 60% MAX Reach: _______ (Paretic)

One button: 80% MAX Reach: _______ (Paretic)

One button: 100% MAX Reach: ______ (Paretic)

L button ____ R button ____ (Paretic)

3) Unilateral Paretic / SRT Reaching Test (10 trials x 3 blocks: **reach in closed fist**)

60% MAX Reach: NDI file names: ______________ E-prime file name: ____________

80% MAX Reach: NDI file names: _______________ E-prime file name: ____________

100% MAX Reach: NDI file names: ______________ E-prime file name: ____________

4) Instruct patients: “When one of the target lights comes on, quickly reach out to touch that target and then return to the start position.”

5) Unilateral Paretic CRT Reaching Test with **disruption** (80% MAX Reach, **reach in closed fist**)

10 blocks of 10; 3 w/o TMS, 6 w/ TMS (150, 200, 250 ms), 1 catch (ChoiceRT_Paired Pulse)

| E-Prime | NDI | Block | Rest (Y/N) | Circle bad trials | | | | | | | | | | | |
| --- | --- | --- | --- | --- | --- | --- | --- | --- | --- | --- | --- | --- | --- | --- | --- |
|  |  | **1** |  | 1 | 2 | 3 | 4 | 5 | 6 | 7 | 8 | 9 | 10 |  |  |
|  |  | **2** |  | 11 | 12 | 13 | 14 | 15 | 16 | 17 | 18 | 19 | 20 |  |  |
|  |  | **3** |  | 21 | 22 | 23 | 24 | 25 | 26 | 27 | 28 | 29 | 30 |  |  |
|  |  | **4** |  | 31 | 32 | 33 | 34 | 35 | 36 | 37 | 38 | 39 | 40 |  |  |
|  |  | **5** |  | 41 | 42 | 43 | 44 | 45 | 46 | 47 | 48 | 49 | 50 |  |  |
|  |  | **6** |  | 51 | 52 | 53 | 54 | 55 | 56 | 57 | 58 | 59 | 60 |  |  |
|  |  | **7** |  | 61 | 62 | 63 | 64 | 65 | 66 | 67 | 68 | 69 | 70 |  |  |
|  |  | **8** |  | 71 | 72 | 73 | 74 | 75 | 76 | 77 | 78 | 79 | 80 |  |  |
|  |  | **9** |  | 81 | 82 | 83 | 84 | 85 | 86 | 87 | 88 | 89 | 90 |  |  |
|  |  | **10** |  | 91 | 92 | 93 | 94 | 95 | 96 | 97 | 98 | 99 | 100 |  |  |

**Adverse Events: Y / N (if yes, explain ___________________________________________________________________)**

**Comments: ________________________________________________________________________________________**

**____________________________________________________________________________________________________________________________________________________________________________________________________**
